# Inferring evolutionary trajectories from cross-sectional transcriptomic data to mirror lung adenocarcinoma progression

**DOI:** 10.1371/journal.pcbi.1011122

**Published:** 2023-05-25

**Authors:** Kexin Huang, Yun Zhang, Haoran Gong, Zhengzheng Qiao, Tiangang Wang, Weiling Zhao, Liyu Huang, Xiaobo Zhou

**Affiliations:** 1 School of Life Science and Technology, Xidian University, Xi’an, China; 2 West China Biomedical Big Data Centre, West China Hospital of Sichuan University, Chengdu, China; 3 Center for Computational Systems Medicine, School of Biomedical Informatics, The University of Texas Health Science Center at Houston, Houston, Texas, United States of America; University of Pittsburgh, UNITED STATES

## Abstract

Lung adenocarcinoma (LUAD) is a deadly tumor with dynamic evolutionary process. Although much endeavors have been made in identifying the temporal patterns of cancer progression, it remains challenging to infer and interpret the molecular alterations associated with cancer development and progression. To this end, we developed a computational approach to infer the progression trajectory based on cross-sectional transcriptomic data. Analysis of the LUAD data using our approach revealed a linear trajectory with three different branches for malignant progression, and the results showed consistency in three independent cohorts. We used the progression model to elucidate the potential molecular events in LUAD progression. Further analysis showed that overexpression of BUB1B, BUB1 and BUB3 promoted tumor cell proliferation and metastases by disturbing the spindle assembly checkpoint (SAC) in the mitosis. Aberrant mitotic spindle checkpoint signaling appeared to be one of the key factors promoting LUAD progression. We found the inferred cancer trajectory allows to identify LUAD susceptibility genetic variations using genome-wide association analysis. This result shows the opportunity for combining analysis of candidate genetic factors with disease progression. Furthermore, the trajectory showed clear evident mutation accumulation and clonal expansion along with the LUAD progression. Understanding how tumors evolve and identifying mutated genes will help guide cancer management. We investigated the clonal architectures and identified distinct clones and subclones in different LUAD branches. Validation of the model in multiple independent data sets and correlation analysis with clinical results demonstrate that our method is effective and unbiased.

## Author summary

Lung adenocarcinoma (LUAD) is a deadly tumor that remains approximately 15% survival rate in 5 years. The diverse reasons that made cancer progression and metastasis include genetic mutation, gene expression alteration, and so on. Understanding this dynamic process and identifying pivotal molecular events driving tumor progression is essential for improving LUAD diagnosis and treatment. Studying time-series data can simulate cancer evolution and determine the temporal patterns of molecular alterations. Unfortunately, it is difficult to collect complete time-series data from individual patients due to various reasons. In this study, we developed a trajectory model based on gene expression of LUAD patients and identified distinct progression branches. Moreover, we found a key gene BUB1B that could lead to aberrant spindle assembly checkpoint (SAC) signaling, and this appeared to be one of the key factors promoting LUAD progression. The trajectory shows clear evident mutation accumulation and clonal expansion along with the LUAD progression. We also investigated the clonal architectures and identified distinct clones and subclones in different LUAD branches. This clones and subclones can be used to identify combination therapy for LUAD. Our results demonstrate the biological utility and clinical application prospects of this progression model.

## Introduction

Lung adenocarcinoma (LUAD) is a deadly tumor that remains approximately 15% survival rate in 5 years [1]. Efforts made for the understanding of LUAD development found that molecular alterations, such as somatic mutation and altered gene expression, play important roles in lung carcinogenesis [2]. Moreover, the accumulation and interaction of molecular alterations made LUAD become a dynamic evolutionary process [3]. Understanding this dynamic process and identifying pivotal molecular events driving tumor progression is essential for improving LUAD diagnosis and treatment.

Studying time-series data can simulate cancer evolution and determine the temporal patterns of molecular alterations [4]. Unfortunately, it is difficult to collect complete time-series data from individual patients due to various reasons. Consequently, large-scale time-series data in human cancer progression studies are rarely available. Therefore, most studies related to cancer progression often limited to mouse models or cell lines. Constrained by sampling limitation, researchers turned to infer human cancer progression by using pseudotime or trajectory inference methods based on cross-sectional data [5,6]. Cross-sectional data come from many cancer patients, mostly from biopsies of untreated tumor samples at the time of diagnosis. The time of onset or diagnosis of these patients varies [7]. Hence, cross-sectional sampling of a single patient can provide a “snapshot” of a specific stage of cancer. Trajectory analysis can extract latent temporal sequences from cross-sectional samples, making it possible to study dynamic biological progression without explicit time-series data [5].

**Fig 1:**
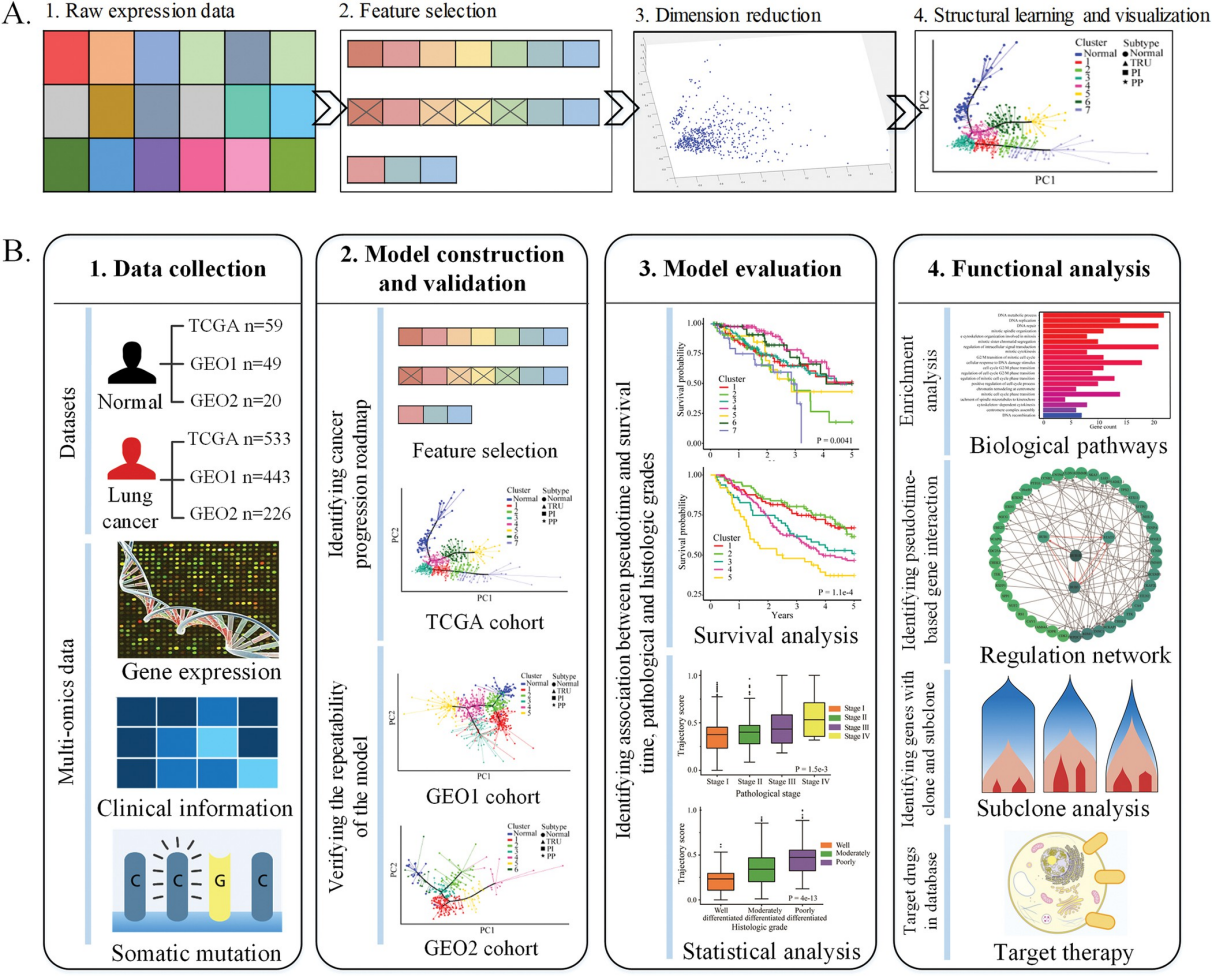
The workflow of the reversed graph embedding and this study. **A.** shows the major steps of the model construction. After the collection of the expression data (Step 1), the feature selection was performed to identify genes involved in LUAD progression (Step 2). Once the progression-related genes were selected, PCA was used for dimensionality reduction and visualization (Step 3). Finally, we used the reversed graph embedding methods to learn and to visualize the underlying structure of the data (Step 4). The obtained structure is referred as the LUAD progression. **B.** shows the workflow of the whole study. It describes the used LUAD cohorts, model construction and validation, model evaluation and biological functional analysis for the model in this study. The data collection panel shows the number of LUAD samples and controls from different sources. We constructed progression model and elucidated the potential molecular events by using multi-omics data. The functional analysis panel shows the enrichment analysis, regulation network analysis and clonal architecture analysis based on the progression model.

With the rapid development of gene expression profiling techniques, transcriptomic data can provide a global assessment of molecular alteration [8]. Patients with different stages of LUAD may harbor molecular processes that reflect disease progression. In this study, we developed a computational approach to infer the progression of LUAD by combining cross-sectional transcriptomic data and machine learning algorithms. To understand the intricate structure of multidimensional data, we employed the powerful technique of reversed graph embedding. In light of previous research, which commonly assumes a tree-like structure for the evolutionary trajectory of tumors, we utilized this algorithm to learn the minimum spanning tree that could best capture the molecular evolutionary path of tumors. As shown in Fig 1A, this intricate model was carefully crafted through a series of rigorous steps, including the selection of LUAD-related genes, modelling LUAD progression with reversed graph embedding, calculation of the trajectory score for each patient, and finally, estimation and validation of the LUAD progression model.

The results show that three different progression branches were identified in LUAD using the Cancer Genome Atlas (TCGA) data. To demonstrate the model’s validity, we applied our approach to two other independent LUAD datasets. The analysis results from these two datasets were consistent with the dataset used for constructing the model. In order to identify the key molecular events responsible for malignant progression in LUAD, we constructed a gene regulatory network by combining the trajectory information and gene expression profiles and identified important regulatory molecules. Moreover, tumor initiation and progression are strongly influenced by genetic variations [9]. We also tested whether genetic variations identified in previous genome-wide association analysis (GWAS) studies were associated with inferred cancer trajectories. The whole workflow is shown in Fig 1B.

Our result showed that several loci, such as PARVA locus and NPAS3 locus, were also mentioned in large-scale lung cancer GWAS study [10]. Furthermore, the trajectory showed evident mutation accumulation and clonal expansion along with the LUAD progression. Extensive studies suggest the diversity of clonal architectures drives cancer evolutionary and intra-tumor heterogeneity, which poses a significant challenge to personalized treatment [3]. Multi-region whole-exome sequencing of LUAD patients showed different subclones which evolved following a branched pattern [11]. Here, we investigated the clonal architecture and subclone selection in three branches to explore the underlying biological mechanisms of LUAD progression and intra-tumor heterogeneity. We also investigated the targeted drugs based on clone and subclone genes for different LUAD branches. In summary, we developed a promising tool to simulate the progress of LUAD and identified potential candidates important for the development of LUAD, thereby providing a scientific basis for LUAD management.

## Results

### Inferring LUAD progression trajectory

**Fig 2:**
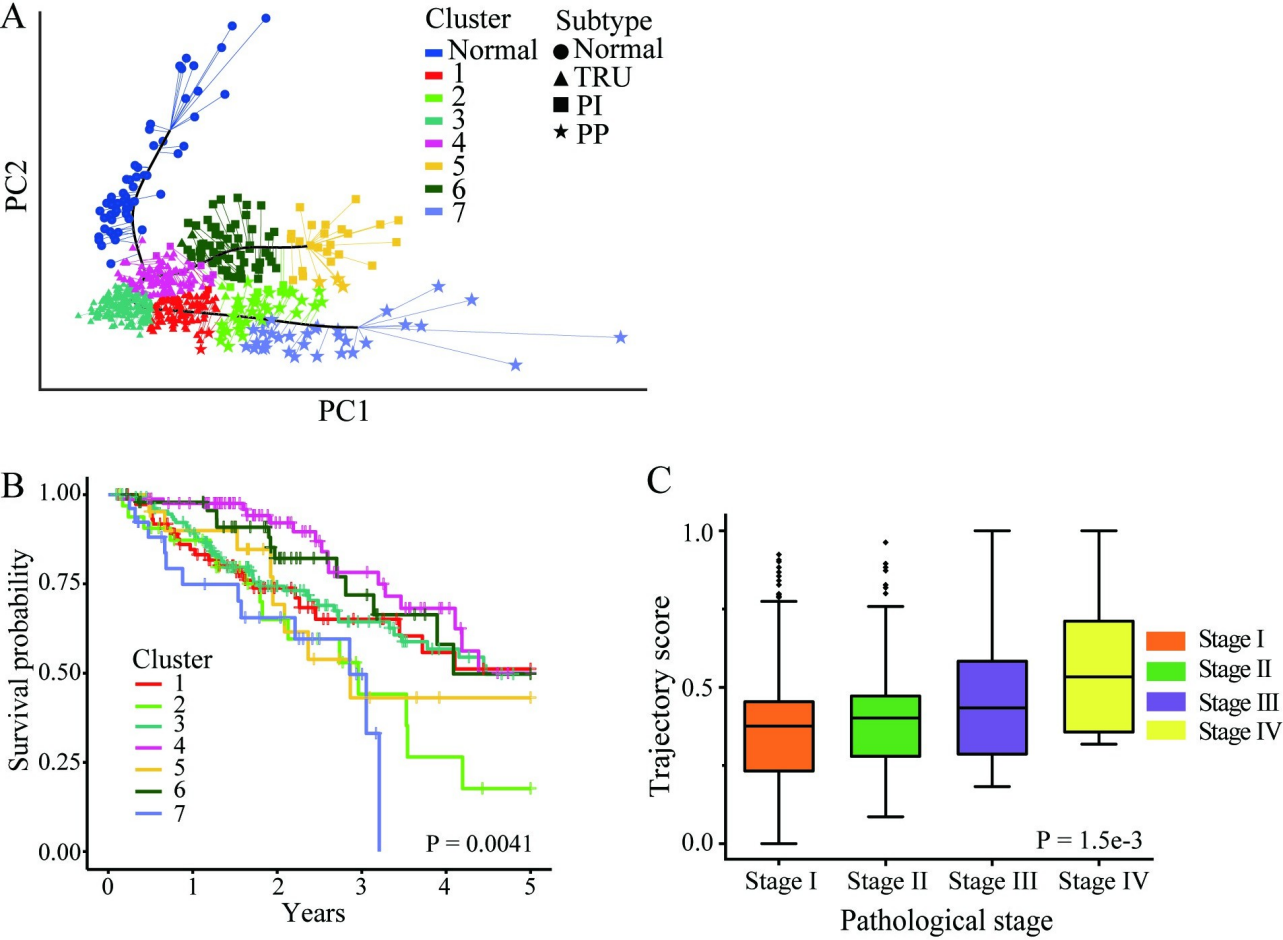
Construction of the progression model in TCGA-LUAD cohort. **A** showed the progression constructed by using 314 candidate genes in the TCGA-LUAD dataset. The triangles, squares and stars represent the molecular subtype of LUAD. The molecular subtypes were identified using 314 candidate genes. The subcluters used for survival analysis are showed in different colors. **B** showed K-M survival analysis among different subclusters. The significance level was calculated using Log-rank test. **C** showed the result of ANOVA analysis among different pathological stages in three independent cohorts. The significance level was set as P<0.05.

The LUAD progression trajectory was constructed by using the TCGA-LUAD dataset. The gene expression data comprises 56,493 genes from 533 primary LUAD cancer samples and 59 normal samples. Firstly, we used minimum redundancy maximum relevancy (MRMR) and incremental feature selection (IFS) methods to select progression-related genes [12,13]. 314 candidate genes related to LUAD progression were identified. Detailed information for these genes is shown in Table B in S1 Appendix. Then, we performed principal component analysis (PCA) for feature dimensionality reduction based on the selected features [14]. We used the three-dimensional features to infer the LUAD progression by using reversed graph embedding [15]. As shown in Fig 2A, Fig A in S1 Appendix and S2 Appendix, by projecting all samples onto the three-dimensional space, we can see that the samples formed three different linear trajectories. The trajectory started from normal samples (blue dots) and first passed through the TRU subtype (terminal respiratory unit, bronchioid, triangles in Fig 2A), then diverged to three branches. One of the terminals remains TRU subtype; the trajectories of the other two branches gradually transited to the PP (proximal-proliferative, magnoid, stars in Fig 2A) or PI (proximal-inflammatory, squamoid, squares in Fig 2A) subtype. Then, we identified seven subclusters by using K-means and gap statistic method [16], which were distributed clearly along the trajectory (seven subclusters shown in different colors in Fig 2A). We investigated the relationship between survival probability and identified progression paths. Fig 2B showed the result of survival analysis among subclusters. We observed significant differences in the survival outcomes among different subclusters (P = 0.0041). The result showed a clear trend of worsening survival outcomes along the paths. For example, along the path from the normal to the PI subtype (subcluster 4 through subcluster 6 to subcluster 5), the survival probability is deteriorating in these subclusters (Fig 2B). Similar results were also observed on other branches. It is worth mentioning that different branches represent independent progression paths of LUAD. Hence, it is difficult to compare survival differences between subclusters on different branches. Here, we only analyzed survival differences between subclusters on the same branch.

To examine the relationship between the clinical outcomes and the progression paths, we calculated the trajectory score for each patient (ranging from 0 to 1). Comparing the trajectory score of different pathological staging groups, the higher the tumor stage, the higher the trajectory score (Fig 2C). There are significant differences between stage groups (significance level P = 1.5e-3). Moreover, significant differences in trajectory scores among different pathological stage groups were also observed in all three branches (see Fig D in S1 Appendix for detailed information). These results indicated that patients with different pathological stages have distinct locations on the progression trajectory.

### Validation of the progression model

**Fig 3:**
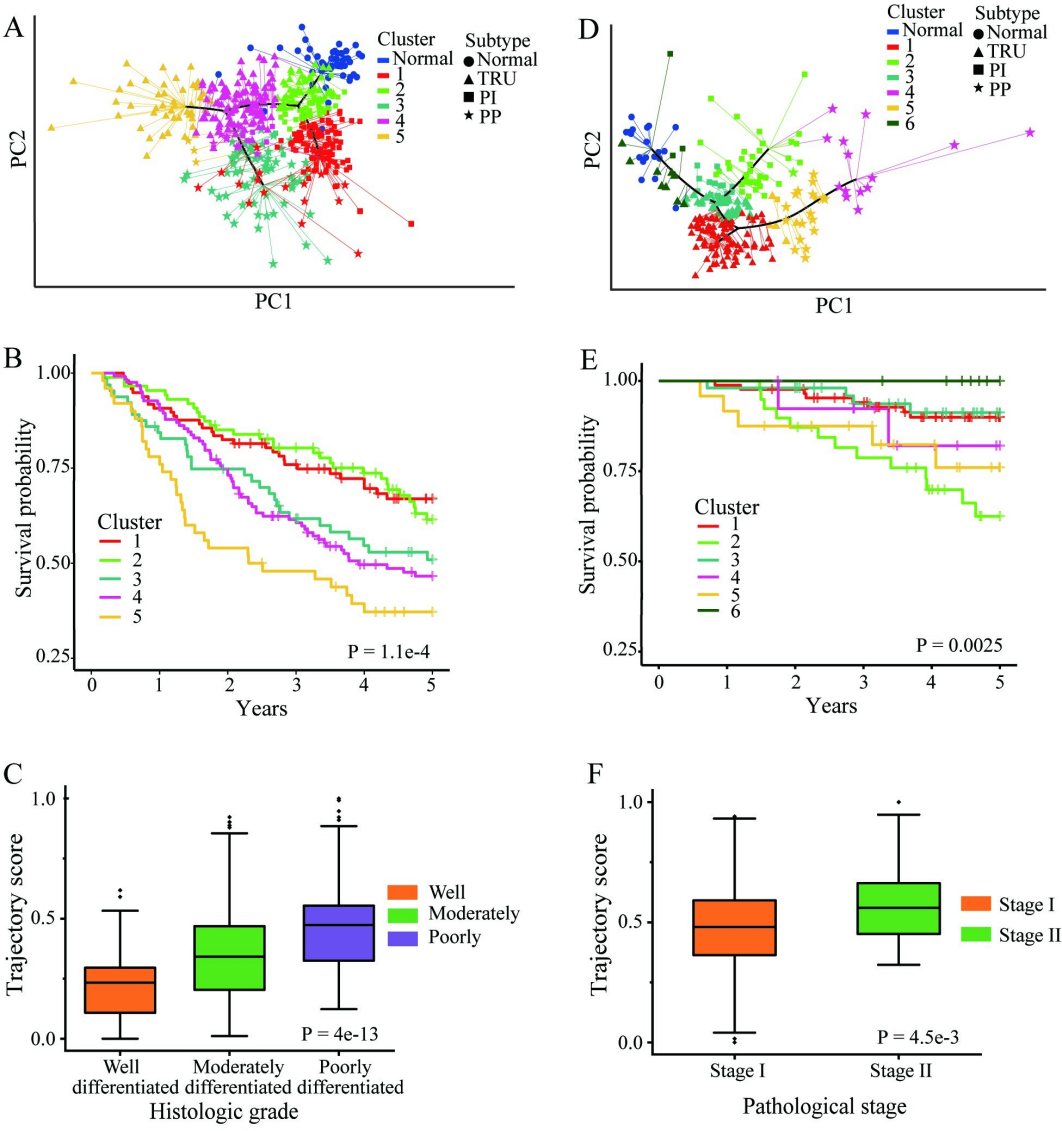
Validation of the progression model in two validation cohorts (GEO datasets). **A** and **D** showed the progression model in cohort 1 and 2, respectively. The subcluters used for survival analysis are showed in different colors. **B** and **E** showed K-M survival analysis among different subclusters. **C** and **F** showed comparison analysis among different histological or pathological stages in cohort 1 and 2, respectively. The significant level was set as P<0.05.

After the same process steps including feature selection and PCA, we applied our methods to the other two independent LUAD cohorts (cohort 1: GSE68465+GSE10072, cohort 2: GSE31210) to validate our progression model [17–19]. Fig 3A, 3D, and Fig B-C in S1 Appendix show the trajectory analysis of validation cohorts 1 and 2, respectively. We can see the trajectories for both cohorts start from the normal samples, pass through TRU subtype, and then diverge to three major branches, including TRU (normal to TRU), PP (normal through TRU to PP) and PI (normal through TRU to PI). Five and six subclusters were identified in cohort 1 and cohort 2, respectively (Fig 3A and 3D). Fig 3B and 3E show the survival analysis of the subclusters identified from the validation cohorts. Survival outcomes among different subclusters are significantly different.

We compared the trajectory scores in different histologic groups in cohort 1. The result shows that the trajectory scores in the three histologic groups are significantly different (Fig 3C). Fig 3F shows the comparison results between the different pathological stages in cohort 2. We also did a comparative analysis of the various branches of each validation cohort, and the results are shown in Fig D in S1 Appendix.

In short, applying our progression model to three independent cohorts results in similar LUAD progress patterns. In addition, our predicted tumor progression trajectory distributions are consistent with patients’ survival probability and pathologically defined tumor stages in the clinic.

### Biological pathways based on progression-related genes

**Fig 4:**
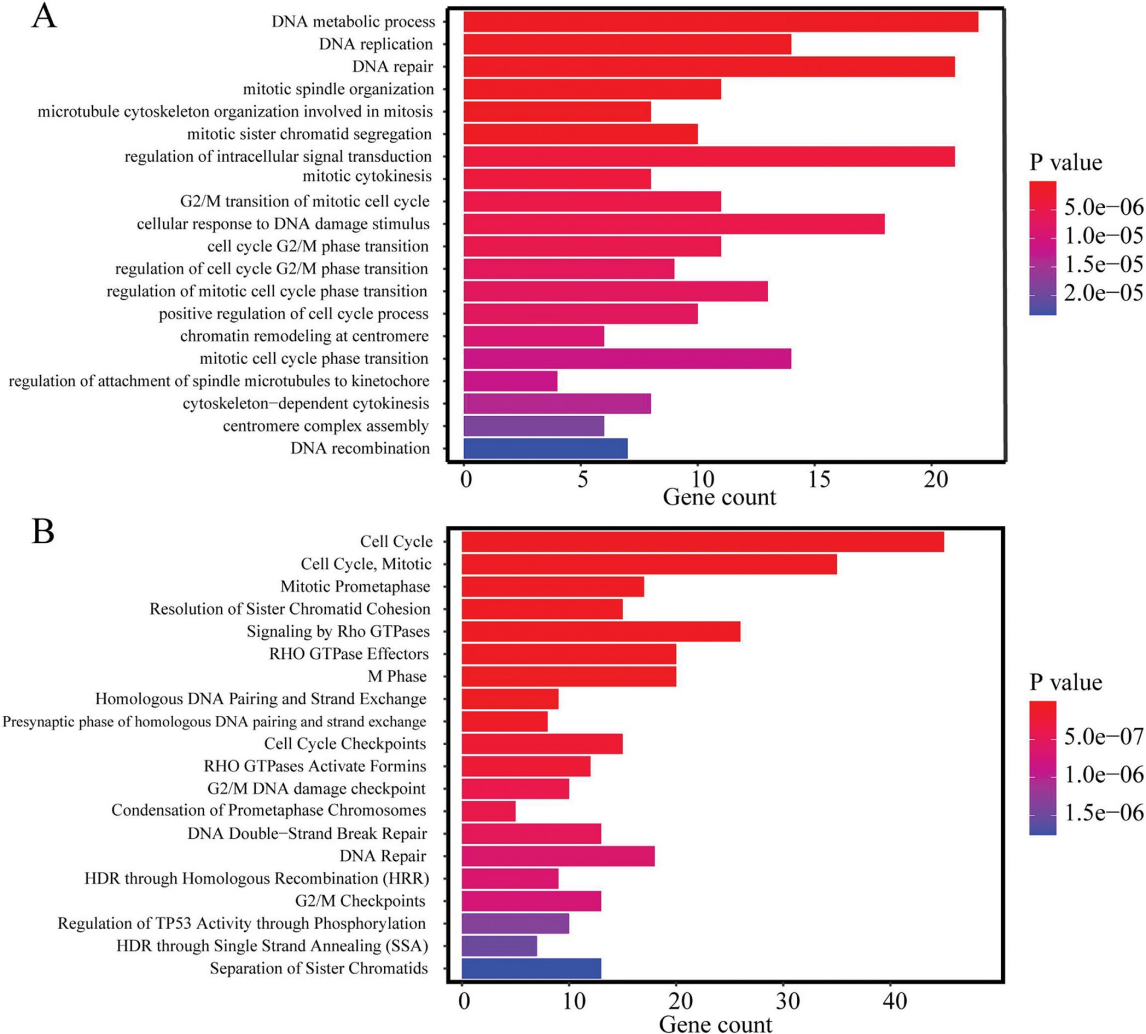
Enrichment analysis for the selected 314 genes by Enrichr. **A** showed the pathways annotated by GO database, and **B** showed the pathways annotated by Reactome database. The significant level was set as P<0.05.

The results of gene ontology (GO) enrichment analysis based on two databases are shown in Fig 4A and 4B.

The top-ranked enriched biological processes include cell cycle, mitosis, and DNA replication, damage, and repair. Cell cycle and mitosis process, including 12 pathways from GO database, such as mitotic spindle organization pathway, mitosis and mitotic sister chromatid segregation pathway (Fig 4A). The result from Reactome also showed several and mitosis process-related pathways, as shown in Fig 4B. The results showed that the biological functions of LUAD progression-related genes are mostly enriched in the pathways related to the cell cycle and mitosis process, suggesting that the cell cycle and mitosis process may be an important factor affecting the biological changes of LUAD progression.

### Gene regulatory network based on LUAD progression

**Fig 5:**
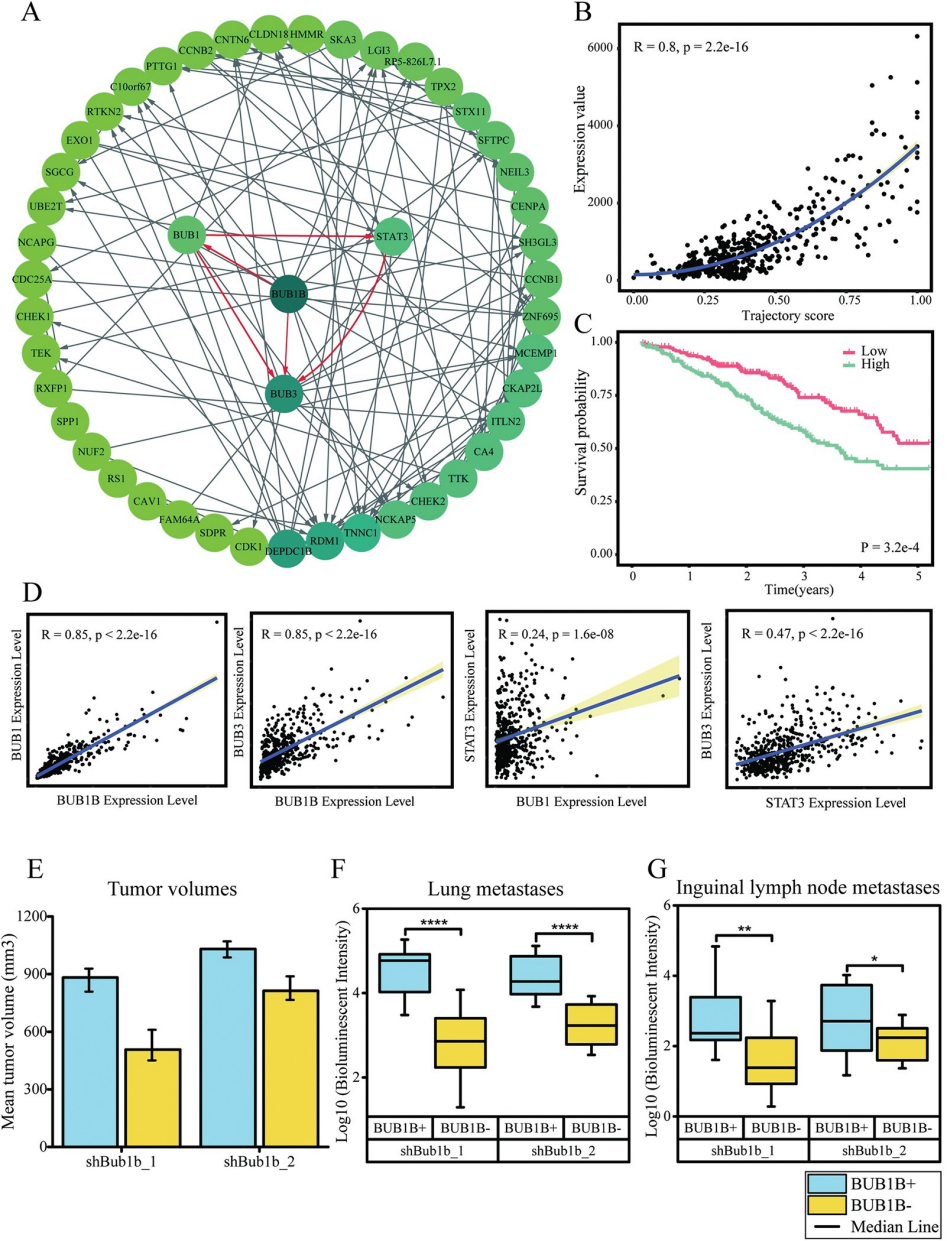
Results of gene regulation network (GRN) based on pseudotime. A showed the whole GRN. The relationship between hub gene BUB1B, BUB1, BUB3 and STAT3 showed with red lines. B showed the association between the expression level of BUB1B and the pseudotime. C showed the survival analysis for high and low BUB1B expression group. D showed the association between the expression of BUB1B, BUB3, BUB1 and STAT3. E showed mean tumor volume in mice implanted with LKPH2 cells after 20 days in two cohorts. Mean±SD is shown in this Fig F and G showed quantification of lung metastases and lymph node metastases measured by ex vivo bioluminescence imaging. BUB1B+ means BUB1B expression group while BUB1B- means BUB1B knockdown group. Each cohort includes 10 tumors samples. (The data of E, F and G extracted from Chen et.al [20]).

Gene regulatory network (GRN) was constructed based on the 314 selected genes using trajectory score as temporal information. The inferred GRN is shown in Fig 5A. The green circles represent genes, and the lines represent the regulatory relationship of the genes. The result indicated that there are complex regulations and interactions between genes during LUAD progression. The more the regulatory relationship of a gene, the darker the color of the circle. BUB1B has more regulatory relationship than other genes and could be regarded as a “hub gene”. We investigated the BUB1B expression along the progression path. As shown in Fig 5B, with the progression of LUAD, the expression of BUB1B gradually increased. We then divided the LUAD patients into two groups (n = 269 in low-BUB1B group, n = 264 in high-BUB1B group) using the median of BUB1B expression value. Survival analysis shows high BUB1B expression level is significantly associated with higher mortality risk (P = 3.2e-4) (Fig 5C).

According to our analysis result, 15 genes can be regulated by BUB1B, such as BUB1, BUB3, TNNC1, SH3GL3 and CDK1. BUB1B, BUB1 and BUB3 are central components of the spindle assembly checkpoint (SAC) in mitosis. Altered expression of SAC genes was observed in many solid tumors and knocking down BUB1B resulted in significantly increased cell death in LUAD cancer cells [20,21]. A previous study found that impaired SAC function plays an important role in many cancers [21]. We found the regulatory relationship of BUB1B, BUB1 and BUB3. We also found that BUB1 can regulate the expression of STAT3, and STAT3 can, in turn, regulate BUB3 expression.

To verify whether BUB1B, BUB1, BUB3 and STAT3 have expression association in LUAD progression, we did a correlation analysis of these genes (Fig 5D). Our analysis indicates that BUB1B is significantly associated with the expression of BUB1 (R = 0.85, P < 2.2e-16) and BUB3 (R = 0.85, P < 2.2e-16). STAT3 is significantly associated with the expression of BUB1 (R = 0.24, P = 1.6e-8) and BUB3 (R = 0.47, P < 2.2e-16).

### Identification of genetic variations associated with LUAD progression

In order to test whether inferred trajectory can provide a quantitative measure in identifying genetic factors associated with LUAD progression, GWAS was performed for LUAD patients in the TCGA cohort. Several single nucleotide polymorphisms (SNPs) presented significant associations with trajectory scores (S1 Appendix Table C). The locus at PARVA gene (rs10734200, significant level P = 6.69e-11) had the most significant association with trajectory scores. PARVA locus was found to associate with LUAD susceptibility in previous large-scale GWAS study [10]. In vitro and in vivo analysis showed that PARVA increased tumorigenicity and metastasis in lung cancer [22]. Some known lung cancer susceptibility loci were also showed association with trajectory scores, such as NPAS3 locus (rs7154051, significant level P = 1.19e-07), DSCAM locus (rs1569094034, significant level P = 5.54e-07) and VLDLR-AS1 locus (rs7029746, significant level P = 1.15e-06) [23].

### Clonal architecture heterogeneity in LUAD progression

We first observed the association between the number of mutation genes and the trajectory scores. The significant positive correlation indicated the existence of mutation accumulation along the progression path of LUAD (Fig E(A) og S1 Appendix). Moreover, in order to understand the clonal expansion during LUAD progression, we first analyzed the relationship between the number of subclones and the progression of LUAD. As shown in Fig E(B) of S1 Appendix, we observed a significant increase of subclone numbers along with the pseudotime progression (P = 1.1e-6). We also found that the number of patients varies with the number of subclones. When the number of subclones is less than 3, the number of patients increases as the number of subclones increases. When the number of subclones is greater than 3, the number of patients decreases as the number of subclones increases (see Fig E(C) in S1 Appendix). Three subclones appear to be more common in LUAD patients, according to our analysis.

**Fig 6:**
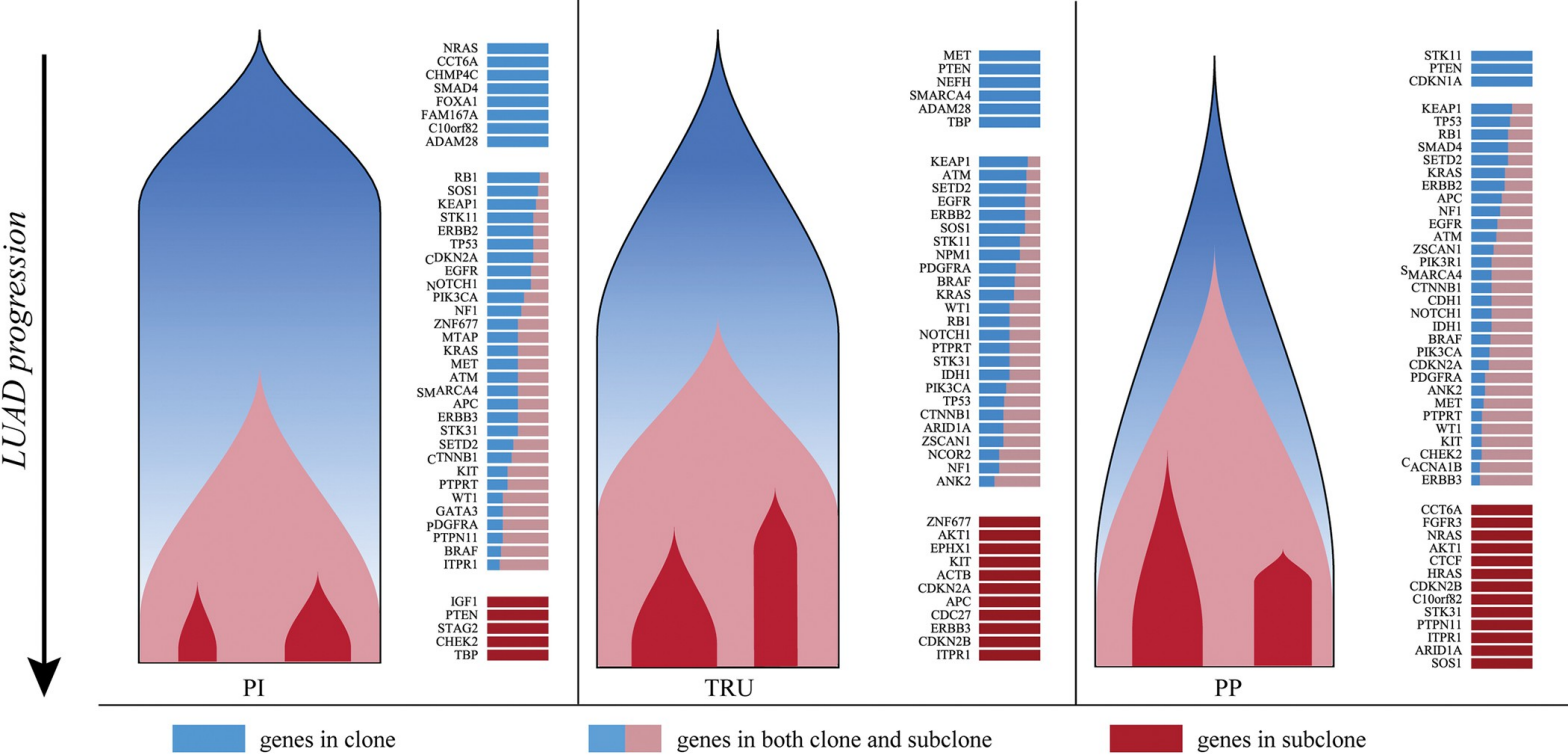
A diagram of clonal architectures of three different LUAD branches. The Fish plots show that the distinct clonal architecture of three different LUAD branches. The bars showed the clonal proportion in genes, and the height and color of the bars correspond to the frequency and genomic characteristics of that clone. Blue bars mean that the genes are in completely clones and are showed in the early stage, orange and blue bars mean that the genes are in both clones and subclones and are showed in the middle stage. Red bars mean that the genes are in completely subclones and are showed in the late stage. Clonal architectures with all genes showed in Fig F-H in S1 Appendix.

Then, we quantified the number and content of LUAD clones and subclones carrying driver mutations in different progression branches. The number of mutated genes is different in the three branches (Fig 6). Ninety-five genes with mutations were found in the normal-TRU branch. 104 and 113 genes with mutations were found in the normal-PI and normal-PP branches, respectively. Moreover, heterogeneous clonal architectures were observed in three LUAD branches. The normal-PI branch contained more genes in the clone (blue) and fewer genes in subclones (red), whereas the situation in the normal-PP branch was opposite. The proportion of genes in subclones in the normal-TRU branch was similar to that in the normal-PP branch, but the normal-TRU branch comprised more genes in the clone. As shown in Fig 6 and Fig F-H in S1 Appendix, the genes that constitute clones and subclones were also different in three branches. For example, mutations in EGFR are predominantly clonal and appear early for both the normal-PI and normal-TRU branches, but not for the normal-PP branch. Some other cancer-related genes such as ERBB2, KRAS, STK11, KEAP1 and TP53 are also mainly included in clones, implying their potential role in tumor initiation. Some genes such as KIT, ITPR1, and DDR2 are primarily subclonal for LUAD progression. These genes may have a greater impact on tumor maintenance.

### Treatment strategies based on clonal architectures

**Fig 7:**
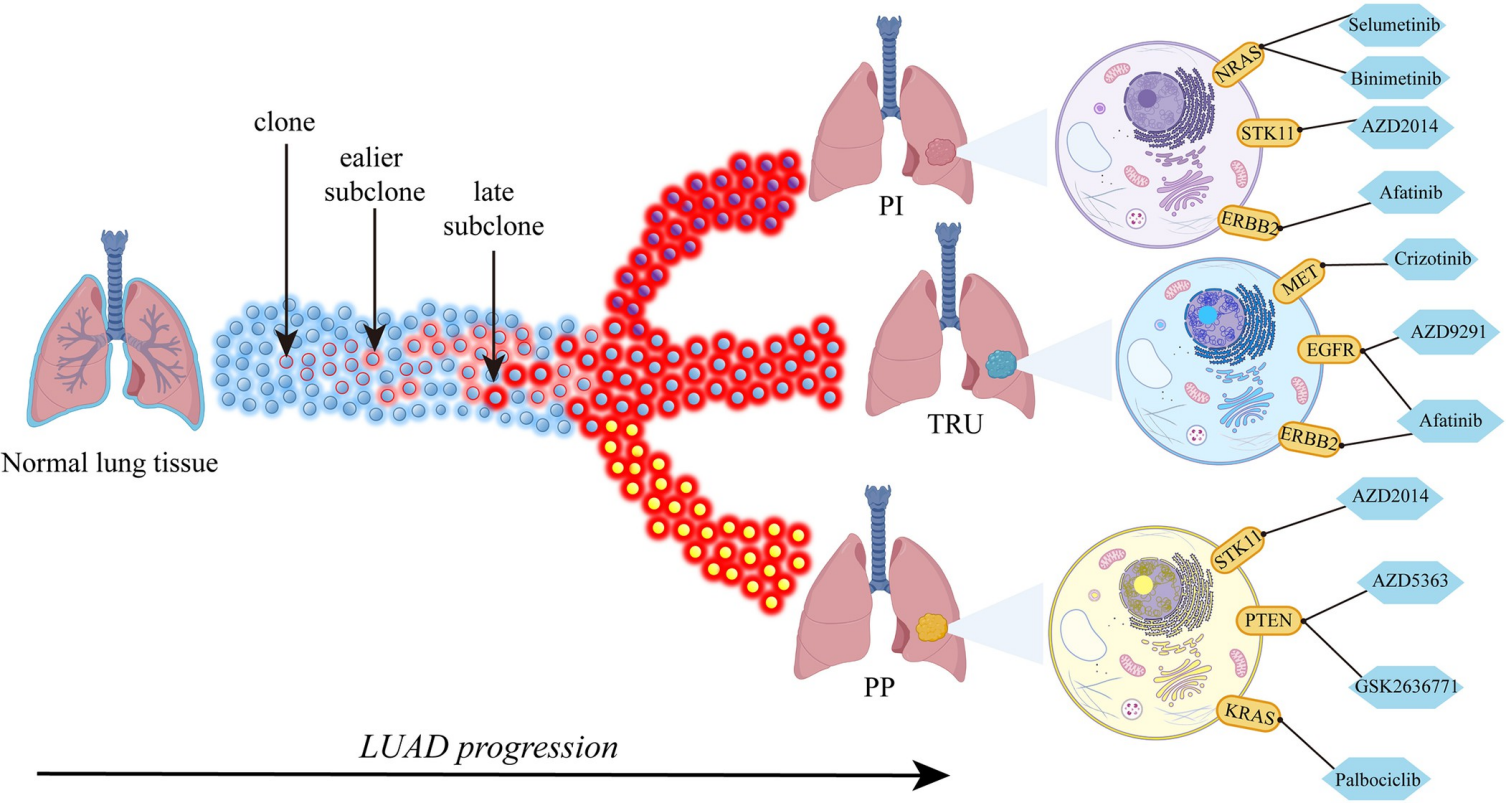
Diagram for the strategies of distinct combination therapy according to distinct clonal architectures. The whole list of drugs and target genes showed in Table D in S1 Appendix. Different therapies should be considered for the intra-tumor heterogeneity. Meanwhile, combination therapies should be adjusted with cancer progression. The diagram elements for lungs and cells were created by using BioRender.com.

We identified 13 of 153 driver genes with their target drugs that had been studied by National Cancer Institute Molecular Analysis for Therapy Choice (NCI-MATCH) and National Lung Matrix Trial (NLMT) [24,25]. The available drugs targeting clonal and subclonal genes are listed in Table D in S1 Appendix. As shown in Fig 7, we proposed the strategies of combination therapies according to distinct clonal architectures. For example, Selumetinib and Binimetinib targeting NRAS may be useful to patients in the normal-PI branch in the early cancer stage. In the middle or late LUAD stage, Trametinib and Vismodegib would produce more-durable responses than other drugs. For patients in the normal-TRU branch, combined treatment with Crizotinib and Afatinib is suggested according to our results.

## Discussion

Since cancer progression and evolution theory was proposed decades ago, extensive studies have significantly expanded our understanding of the theory [3,26]. However, due to the lack of time-series data, very few studies cover the entire cancer progression based on large sample size. To our knowledge, few studies have estimated molecular processes in lung cancer. The computational approach that we developed in this study has advantages in overcoming sampling limitations and inferring cancer progression paths from cross-sectional transcriptomic data. We applied this approach to large-scale LUAD datasets and identified a linear trajectory with three different branches. One branch directly transited to the TRU-like subtype, and the other two branches first passed through the TRU-phenotype subtype and then gradually transited to the PP-phenotype or PI-phenotype subtype. Combining the results of previous studies and our analysis, we speculate that the TRU subtype may be the earlier stage of LUAD [27]. Furthermore, the validation in independent cohorts and the association analysis of survival data and pathological stages support the validity of the progression model.

Currently, the key molecular events that are responsible for malignant progression in LUAD are not well understood. Enrichment analysis of progression-related genes revealed that most of the pathways were related to the cell cycle, mitosis process, and DNA replication, damage, and repair. We constructed a gene regulatory network based on pseudotime to analyze interactions between progression-related genes and to identify key factors in LUAD progression. The result showed BUB1B gene is considered as a “hub gene” with more regulatory relationship than other genes. BUB1B plays a critical role in mitotic checkpoint signaling and stable attachment of kinetochores to spindle microtubules [28]. A previous study found that BUB1B was overexpressed in several LUAD cell lines, as shown in Table E in S1 Appendix [20]. Knockdown of BUB1B significantly reduced the LUAD cell growth and soft agar colony formation [20]. BUB1B is an important factor for tumor cell growth, proliferation and metastasis [20]. Previous study indicated tumor implantation in mice showed that inhibition of BUB1B reduced tumor volumes by approximately 30% in two independent cohorts (Fig 5E). Quantification of lung and lymph node metastases based on ex vivo bioluminescence imaging demonstrated a significant decrease in metastases following BUB1B inhibition (Fig 5F and 5G). Moreover, inhibition of BUB1B significantly prolonged the survival of the mice in two cohorts [20]. In addition, inhibition of BUB1B significantly extended the survival time of mice in two sets of experiments. In experiment one, the median survival was 26 days in BUB1B expressing group and 39 days in BUB1B inhibition group (log-rank P< 0.001). The median survival was 27 days in the BUB1B-expressing group and 37 days in the BUB1B inhibition group (log-rank P<0.001) in experiment 2 [20]. These results are consistent with the pseudotime analysis shown in Fig 5B; that is, the expression level of BUB1B increases with the progression of pseudotime and is associated with the overall survival of LUAD patients.

As an important paralogous gene of BUB1B, BUB1 interacts closely with BUB1B in SAC. The physical interaction between BUB1 and BUB1B with blinkin protein is mediated by a conserved N-terminal region [29]. These interactions make BUB1 can cooperate with BUB1B for the kinetochore localization in the mitotic checkpoint. In early prometaphase, BUB1 also plays a critical role in recruiting downstream main checkpoint factors, such as BUB3 and CDC20 [30,31]. BUB1 can also promote the incorporation of these proteins, thereby directly facilitating exit from mitosis [30]. Furthermore, BUB1B and BUB1 could regulate the binding ability of BUB3 to kinetochore phospho-target via conservative motifs called Gle2-binding-sequence (GLEBS). Overexpression of the GLEBS motif could lead to SAC dysfunction by BUB1B and BUB1 competition for binding BUB3 [29]. In addition, the BUB1-BUB3 complex could keep DNA telomere from shortening and fragility, which might provide support for the proliferation of tumor cells [32]. The expression of these genes also showed high correlations in our results (R>0.8, see Fig 5D). Our results supported that changed BUB1B expression could result in the defects of spindle attachment and the impairment of the mitotic checkpoint, which can further lead to mitotic catastrophe in dividing cells and promote tumorigenesis.

On the other hand, we also found the regulatory relationship between BUB1, STAT3, and BUB3. A prior study found BUB1 can promote TGF-beta signaling by interacting with TGF-beta receptors in non-dividing cells [33]. Sudies have shown that TGF-beta promotes tumor cell invasion, metastasis and immune escape [34,35]. TGF-beta stimulates STAT3 expression and induces epithelial-mesenchymal transition in lung cancer cells through activation of STAT3 signaling pathway. Furthermore, the STAT3 can promote microtubule formation via the interaction with stathmin, potentially influencing the SAC cell cycle control. Activation of STAT3 may promote the cell cycle process to accelerate tumor cell proliferation [36]. Our results showed that the pseudotime trajectories might be used to identify key factors promoting LUAD progression.

Extensive studies have shown that in addition to transcriptomic remodeling, carcinogenesis and subsequent neoplastic events are highly dependent on genetic factors in the human genome [9]. Traditional analysis based on case-control methods has disadvantages in assessing the role of genetic variations in cancer progression [37]. Our study showed the opportunity of inferred transcriptomic trajectory to be used as a molecular phenotype to identify progression-related loci. Despite a limited sample size, several loci were identified in previous large-scale GWAS studies showed significant associations with the inferred trajectory. For example, in our study, the variant in PARVA showed significant association with the susceptibility and progression of LUAD. This result showed consistency with previous GWAS study [10]. Prior study also found that PARVA plays a critical role in promoting lung cancer by regulating ILK pathway [22]. However, the function of identified loci in LUAD still need to be validated in future work. Subsequent research can further advance functional interpretation by combining candidate genetic factors with disease progression.

Additionally, LUAD also carries multiple clonal expansions driven by accumulated mutations. The inferred trajectory could reflect increased mutation genes alone the progression path, which showed consistency with prior study [38]. Extensive evidence demonstrated one of the fundamental biological mechanisms underlying cancer evolution is clonal selection and expansion [39]. Moreover, molecular and phenotypic intra-tumor heterogeneity due to clonal diversity is closely related to the progression and evolution of cancer and brings significant challenges to personalized treatment [40]. Most solid tumors are comprised of multiple clones and subclones [39]. Somatic mutations shared by all tumor cells reflect their clonal origin. Clone lineages diverge to form distinct subclones. Subclones harbor additional mutations in subpopulations. Phylogenetic analysis reveals that tumor evolution is branched rather than linear [41]. Our clonal architecture analysis of different LUAD branches predicted by the progression model shows the number of subclones increases with the LUAD progression, resulting in a wide variety of subclones (see Fig E(B) and (C) in S1 Appendix). Our analysis indicates that some well-known driver genes, such as EGFR, TP53, and ERBB2, are involved in the early cloning of LUAD. This result is to some extent consistent with previous evolutionary studies [42]. We also observed the clonal heterogeneity in different branches, as shown in Fig 6 and Fig F-H in S1 Appendix. For example, NRAS is clonal in the normal-PI branch, but is absent or subclonal in other branches. Although many therapeutic approaches are available or under development in recent years, treatment failure or resistance often occurs during chemotherapy [43]. According to our analysis, intra-tumor heterogeneity appears to be the key obstacle for LUAD treatment. Based on clonal architecture analysis of LUAD with different progression patterns, we can see different types of LUAD have distinct gene mutation profiles. Therefore, different treatment methods should be considered according to the genetic characteristics of the tumor. For example, patients in the normal-PI progression path can be treated with Selumetinib or Docetaxel to target NRAS and patients in the normal-PP path can be treated with the driver gene inhibitors in the specific subclones. According to our analysis results, combination therapy adjusted with cancer progression can be a new strategy to combat cancer [44]. The treatment can be targeted to clone genes at the early stage, and then gradually incorporates drugs targeting on both clone and subclone genes. This approach may provide more therapeutic anti-cancer benefits.

In this study, we developed algorithms to model LUAD progression based on cross-sectional data. Our results demonstrated the biological utility and clinical application prospects of this progression model. The predicted results by our progression model provide valuable information and research directions for cancer researches. Based on the genomic testing results from individual patients, the position of a single case on the progression path can be determined and treatment regimens can be proposed. The application of quantitative clinical and molecular data such as clinical outcome, genetic variants, DNA copy number and somatic mutation, provided evidence for the robustness and biological function of this model. In addition, multi-omics data can also be used to improve the resolution of the progression model in understanding the tumor progression and evolution.

In this study, we applied the reversed graph embedding method to model the molecular progression of LUAD, which was validated in multiple independent datasets and found to be effective and unbiased. Our method identified several candidate molecular factors associated with disease progression. The potential of the reversed graph embedding method in inferring pseudotime trajectories has been demonstrated in a previous study on breast cancer [45]. However, our study differs from previous research in that we not only identified the key regulators during lung cancer progression, but also investigated their biological and clinical relevance based on the inferred trajectory. These results suggest that the reversed graph embedding method is unbiased across tumor types and has the potential to infer cancer progression using expression data of other tumor types. However, it is important to note that the trajectories computed by algorithms need to be further confirmed by experiments.

## Methods

### Datasets

This study used gene expression data from four LUAD datasets from TCGA (https://www.cancer.gov/tcga) and Gene Expression Omnibus (GEO) (www.ncbi.nlm.nih.gov/geo/) databases. The gene expression matrix from the TCGA data is derived from bulk RNA-sequencing, while the gene expression matrix from the GEO dataset is obtained from microarray technology. A total of 1202 LUAD tumor samples and 128 normal samples were included in our study. Firstly, gene expression data of 533 LUAD samples and 59 normal samples from TCGA-LUAD dataset were downloaded from the Genomic Data Commons (GDC) Data Portal (https://gdc-portal.nci.nih.gov/) using R package TCGAbiolinks [35]. DNA copy number, somatic mutation and clinical data were downloaded from TCGA data portal (https://portal.gdc.cancer.gov/). TCGA-LUAD dataset was used as the training cohort for model construction (https://www.cancer.gov/tcga). Then, gene expression data of GSE68465 and GSE10072 were obtained from the Gene Expression Omnibus (GEO) database [17, 18]. We combined the 443 LUAD samples from GSE68465 and 49 normal samples from GSE10072 as validation cohort 1. It is worth noting that the gene expression data for these two datasets were generated by using a common platform GPL96 (Affymetrix GeneChip Human Genome U133 Array Set HG-U133A). Thus, the number of genes contained in these two datasets are the same. We used ‘ComBat’ function in the SVA package to avoid batch effects [46,47]. Moreover, gene expression data of 226 tumor samples and 20 normal samples were collected from GSE31210 as validation cohort 2 [19]. Clinical information such as survival time and pathological and histologic stages of these samples was obtained from GEO for downstream analysis. The detailed information of four datasets is shown in Table A in S1 Appendix.

### Gene selection for LUAD progression

For high-dimensional microarray data (including more than 50,000 genes), not all genes bear useful information for cancer progression modeling. Thus, the first step is to identify candidate genes involved in LUAD progression. Previous studies selected features based on cancer stages or survival times [48,49]. However, cancer stages and surviving times are not good feature selection criteria, and they may be influenced by confounding factors such as living environment and drug therapies. In addition, most predictive studies tend to group patients based on their prognosis. Thus, patients with similar clinical outcome have always been grouped together, resulting in distorted progression models. In this study, we used molecular subtypes of LUAD, including TRU, PP and PI, as labels to select progression-related genes [27]. The LUAD molecular subtypes were identified using a 506-gene nearest centroid classifier provided by the previous study [27]. We then applied the MRMR method for feature selection [12]. MRMR could minimize the mutual information among the selected features, while maximizing the mutual information between the selected features and the labels.

Additionally, since MRMR can only provide a list of genes ranked by their relevance and redundancy, it cannot determine the optimal number of selected features. Thus, we used the IFS method to determine the optimal number of the selected features [13]. Jackknife test was used to evaluate the prediction accuracy for each feature set [50]. The one that achieves the highest prediction accuracy is considered as the optimal feature set.

### Modelling LUAD progression by using reversed graph embedding

Once the progression-related genes were selected, PCA was used for dimensionality reduction [44]. In this study, we used the top-ranked three principal components, so that each sample can be projected as a point and be visualized in three-dimensional space.

We used a structure learning framework based on reversed graph embedding to model LUAD progression [15]. Compared with the traditional principal curve approaches, this method can handle self-intersecting data and has shown flexibility in practical applications [15]. In addition, this framework can learn a minimum-cost spanning tree or a weighted undirected *l*_1_ graph. Previous studies on the cancer evolution theory have shown that the trajectory of cancer progression is more likely to be a tree-like structure [39]. Here, we developed the algorithm to learn a minimum-cost spanning tree for modeling LUAD progression. The minimum-cost spanning tree algorithm can be defined as the following minimization problem:

$$\underset{W\in W_{0}}{min}{\sum_{i=1}^{N}\sum_{j=1}^{N}w_{i,j}\|f_{\xi }(y_{i})-f_{\xi }(y_{j})\|}_{2}^{2}$$ (1)

where the set of linear constraints is given by:

$$W_{0}=\{W\geq 0\}\cap W\prime$$ (2)

and *W*′ can be described by the following constraints,

$$W\prime :\{W=W^{T}\}\cap \{\frac{1}{2}\sum_{i,j}w_{i,j}=|V|-1,w_{i,i}=0,\forall i\}\cap \{\frac{1}{2}\sum_{i\in \delta ,j\in \delta }w_{i,j}\leq |\delta |-1,\forall \delta \subseteq V\}$$ (3)

Let $\{x_{i}{\}}_{i=1}^{N}\in R^{D\times N}$ represent the expression data with *N* samples and *D* genes of the input space, $\{y_{i}{\}}_{i=1}^{N}\in R^{d}$ is a set of latent points in lower-dimensional space *d*, *f*_*ξ*_ is a projection function that maps *y*_*i*_ to some points in the original input space, $V=\{V_{i}{\}}_{i=1}^{N}$ is a set of vertexes where each *V*_*i*_ corresponds to a latent point *y*_*i*_, *W*∈*R*^*N*×*N*^ is a matrix with the (*i*, *j*) th element denoted by *w*_*i*,*j*_ which represents the connectivity between *y*_*i*_ and *y*_*j*_, *w*_*i*,*j*_>0 means the edge (*V*_*i*_, *V*_*j*_) exits, and 0 otherwise. The first constraint of *W*′ makes sure the connection of the undirected graph is symmetric, the second constraint means the minimum-cost spanning tree contains |*V*|−1 edges, and the third constraint limits the tree with the properties of acyclicity and connectivity. The input expression matrix $\{x_{i}{\}}_{i=1}^{N}$ the latent space $\{y_{i}{\}}_{i=1}^{N}$, and the projection function *f*_*ξ*_ can be simultaneously optimized based on the Laplacian eigenmap using the formulation below:

$$\underset{\left\{y_{i}\},W,\{p_{i,j}\right\}}{min}{\sum_{i=1}^{N}\sum_{j=1}^{N}w\|f_{\xi }(y_{i})-f_{\xi }(y_{j})\|}_{2}^{2}+\gamma \sum_{i=1}^{N}\sum_{j=1}^{N}p_{i,j}[{\left\|x_{i}-f_{\xi }(y_{i})\right\|}_{2}^{2}+\sigma \;\log\;p_{i,j}]$$ (4)

$$s.t.\sum_{j=1}^{N}p_{i,j}=1,p_{i,j}\geq 0,\forall i,j$$ (5)

where *p*_*i*,*j*_ is the probability of assigning sample *x*_*i*_ to projection point *y*_*j*_. *σ*>0 is a regulation parameter to reduce the error of data reconstruction. The optimization problem given in Eq (4) is a biconvex problem and can be solved by using the alternate convex search [15]. A Fig that illustrating the reversed graph embedding method is provided in Fig I.

### Calculation of trajectory score

The order of LUAD samples on the minimum-cost spanning tree was quantified by trajectory score. We first determine the root point as the starting point of the LUAD progression by finding the vertex of the branch where the normal samples lie on. Then, we projected each sample to its closest point on the minimum-cost spanning tree. We calculated trajectory scores of individual tumor samples based on the distance between the projection point and the root point along the tree. The definition of trajectory score can be summarized as following formulation:

$$Trajectoryscore=L(r,f_{\xi }(y_{tumor}))$$ (6)

*f*_*ξ*_(*y*_*tumor*_) is the projection point of a tumor sample onto minimum-cost spanning tree graph. *r* is the root point on the graph, and L(r,y) is the distance metric between the vertices *r* and the point *y* along the graph. In other words, the trajectory score of a tumor sample is the distance between the root point *r* and the projection point *f*_*ξ*_(*y*_*tumor*_) onto the minimum-cost spanning tree. The trajectory score of each LUAD sample reflects the severity of tumor progression; a lower trajectory score represents the lower tumor grade and better prognosis.

### Model assessment by using survival time and clinical grades

For the training cohort (TCGA cohort) and the validation cohorts (GEO cohorts), the trajectory scores among different pathological and histological grades were compared by using ANOVA analysis, respectively. To investigate the relationship between survival time and identified progression paths, LUAD patients were divided into different subclusters by using K-means algorithm for three cohorts, respectively. We applied the gap statistic method to estimate the optimal number of the subclusters [16]. The method calculates the gap statistic for different numbers of clusters and selects the number of clusters where the gap statistic is the highest. After collection of the survival data for each patient, we can calculate the survival probabilities among different subclusters. We performed a five-year Kaplan-Meier survival analysis across different subclusters for three cohorts, respectively. Log-rank P value was calculated to determine the significant differences in survival outcomes. Survival analysis and ANOVA analysis were conducted using survival and ggpubr packages, respectively [51].

### Enrichment analysis of LUAD progression-related genes

To investigate the critical biological process and functional pathways responsible for the LUAD progression, we performed GO enrichment analysis on the selected LUAD progression-related genes using the Enrichr web tool (https://maayanlab.cloud/Enrichr/) [52]. By input the gene list, Enrichr can assign GO terms to the genes using pathway databases. In this study, the pathway enrichment was based on the Gene Ontology database and Reactome pathway knowledgebase, respectively [53, 54]. Then, it can calculate the number of genes associated with each GO term in the entire genome or a reference set. It can also test whether the number of candidate genes associated with each GO term is significantly higher than expected by chance to obtain the significance level. The significance level was set as P<0.05.

### Construction of gene regulatory network based on trajectory

In order to identify the key molecular events and better understand the underlying biological process in the malignant progression of LUAD, we constructed the GRN by combining trajectory scores and progression-related genes. In our study, AR1MA1-VBEM method was used to investigate the pseudo-temporally regulatory relationship [55]. This method uses the first-order autoregressive moving-average model to fit gene expression data and constructs GRN via a variational-Bayesian framework. The method assumes that each gene in the network is influenced by the expression of other genes, which are represented as nodes in a directed graph. The edges in the graph indicate the regulatory relationships between genes. The AR1MA1-VBEM method first models the gene expression data as a time-series data. Then, it uses a variational Bayes expectation-maximization algorithm to estimate the model parameters and infer the regulatory relationships between the genes. We ran AR1MA1-VBEM algorithm using non-informative priors and set the posterior probability threshold to 0.5. Then we identified significant regulatory relationships by selecting the genetic associations with a weight greater than 0.3 or less than -0.3 to identify the significant regulatory relationship. All settings were held at the default values. The GRN was visualized with Cytoscape 3.7.2 (https://cytoscape.org/) [56].

### Association analysis of single nucleotide variants and progression trajectories

We performed GWAS to identify the candidate loci associated with an inferred trajectory in the TCGA cohort. Genome association and prediction integrated tool (GAPIT) and general linear model (GLM) model were used for this analysis [57]. SNP genotyping for 906,600 target SNPs was downloaded from the TCGA database. Quality control was preprocessed on the subject level using GAPIT; only variants with minor allele frequency (MAF) > 0.01 and linkage disequilibrium decay distance < 0.1 were considered. Total 871,378 SNPs were collected for the downstream analysis. The significant threshold for GWAS analysis was set as P<. A lower p-value indicates a stronger association between the genetic factor and the phenotype, while a higher p-value suggests a weaker association.

### Identification of clonal architectures in different progression branches

To test the associations between inferred trajectories and mutation accumulation and clonal expansion, we performed an association analysis of trajectory scores and number of mutated genes. Moreover, identifying the occurrence order of cancer driver events can help understand which genes are involved in tumor initiation or maintenance. Branched evolution is one of the characteristics of cancer [11]. Thus, we compared clonal architectures among three branches based on a list of potential LUAD driver genes collected from the The Catalogue Of Somatic Mutations In Cancer (COSMIC) cancer gene census. The driver genes were identified in prior large-scale pan-cancer studies and large-scale LUAD studies. For each driver gene, the number of clones and subclones were calculated by using SciClone package [58]. SciClone is a computational method that analyzes the variant allele frequencies (VAF) of somatic mutation data to infer clonal architecture and identify the genetic composition of clones and subclones.

Moreover, the evolutionary framework is an essential guide for potential treatment strategies. We investigated the targeted drugs for the driver genes in two drug studies, including the NCI-MATCH trial and the NLMT. The whole workflow is shown in Fig 1. The model construction is performed using MATLAB (version: 2018a) script, and the downstream analyses, including survival analysis, ANOVA analysis, enrichment analysis, GRN analysis, SNP analysis and subclone analysis are performed using R (version 4.1.0) script.

## Supporting information

S1 Appendix Fig A. Three-dimensional view of the model for TCGA-LUAD. The video is shown in the S2 Appendix. **Fig B.** Three-dimensional view of the model for validation cohort 1. **Fig C.** Three-dimensional view of the model for validation cohort 2. **Fig D.** Pseudotime value showed significant difference in distinct pathological stages for 3 different branches of three cohorts: Normal to PI (A, D, G), Normal to TRU (B, E, H), Normal to PP (C, F, I). (A-C) Three branches for TCGA-LUAD. (D-F) Three branches for validation cohort 1. (G-I) Three branches for validation cohort 2. **Fig E.** (A) Association between trajectory score and the number of mutated genes. (B) The relationship between the number of subclones and the progression of LUAD. The number of subclones significantly increased with the progression of LUAD. (C) The sample number included in different subclone number group. This result showed that 3 subclones may more common in LUAD patients. **Fig F.** The proportion of clone and subclone for genes in normal-PI branch. **Fig G.** The proportion of clone and subclone for genes in normal-TRU branch. **Fig H.** The proportion of clone and subclone for genes in normal-PP branch. **Fig I.** A cartoon illustrating the reversed graph embedding method. **Table A.** Information of the four datasets used in this study. **Table B.** 314 progression-related genes selected by using MRMR and IFS method. **Table C.** SNP loci associated with inferred trajectory based on GWAS analysis. **Table D.** All available targetable genes and target drugs according to NLMT and MATCH. **Table E.** Soft agar colony formation in several human LUAD cell lines. The colony number of human BUB1B siRNA SMRTpool (siBUB1B) were shown as relative values normalized to controls (siNC).
(PDF)

S2 Appendix The video that shows the progression model for TCGA-LUAD. (MP4)

S3 Appendix The video that shows the progression model for validation cohort 1. (MP4)

S4 Appendix The video that shows the progression model for validation cohort 2. (MP4)
