# Supplementary material for: Inferring evolutionary trajectories from cross-sectional transcriptomic data to mirror lung adenocarcinoma progression
Source: PLoS Comput Biol. 2023 May 25;19(5):e1011122. doi: 10.1371/journal.pcbi.1011122 (PMC10246837; doi:10.1371/journal.pcbi.1011122)
Supplement: S1 Appendix — Three-dimensional view of the model for TCGA-LUAD. The video is shown in the S2 Appendix. Fig B. Three-dimensional view of the model for validation cohort 1. Fig C. Three-dimensional view of the model for validation cohort 2. Fig D. Pseudotime value showed significant difference in distinct pathological stages for 3 different branches of three cohorts: Normal to PI (A, D, G), Normal to TRU (B, E, H), Normal to PP (C, F, I). (A-C) Three branches for TCGA-LUAD. (D-F) Three branches for validation cohort 1. (G-I) Three branches for validation cohort 2. Fig E. (A) Association between trajectory score and the number of mutated genes. (B) The relationship between the number of subclones and the progression of LUAD. The number of subclones significantly increased with the progression of LUAD. (C) The sample number included in different subclone number group. This result showed that 3 subclones may more common in LUAD patients. Fig F. The proportion of clone and subclone for genes in normal-PI branch. Fig G. The proportion of clone and subclone for genes in normal-TRU branch. Fig H. The proportion of clone and subclone for genes in normal-PP branch. Fig I. A cartoon illustrating the reversed graph embedding method. Table A. Information of the four datasets used in this study. Table B. 314 progression-related genes selected by using MRMR and IFS method. Table C. SNP loci associated with inferred trajectory based on GWAS analysis. Table D. All available targetable genes and target drugs according to NLMT and MATCH. Table E. Soft agar colony formation in several human LUAD cell lines. The colony number of human BUB1B siRNA SMRTpool (siBUB1B) were shown as relative values normalized to controls (siNC). (PDF) [file pcbi.1011122.s001.pdf]

S1 Appendix

Inferring Evolutionary Trajectories from Cross-sectional Transcriptomic Data to Mirror Lung Adenocarcinoma Progression- Supplementary Material

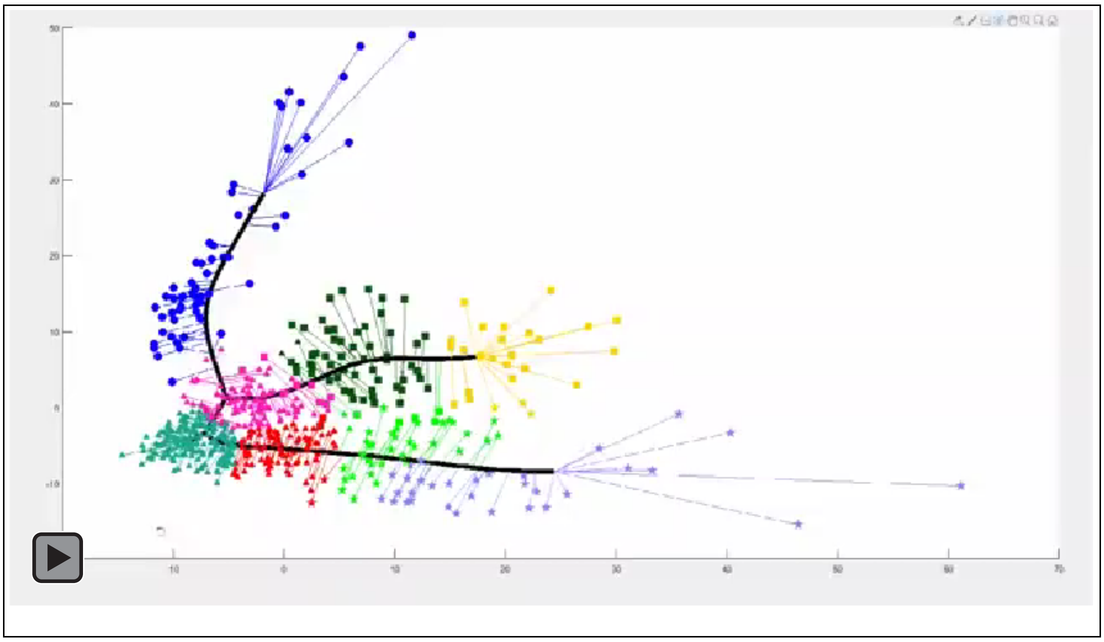

Fig A. Three-dimensional view of the model for TCGA-LUAD. The video is shown in the S2 Appendix.

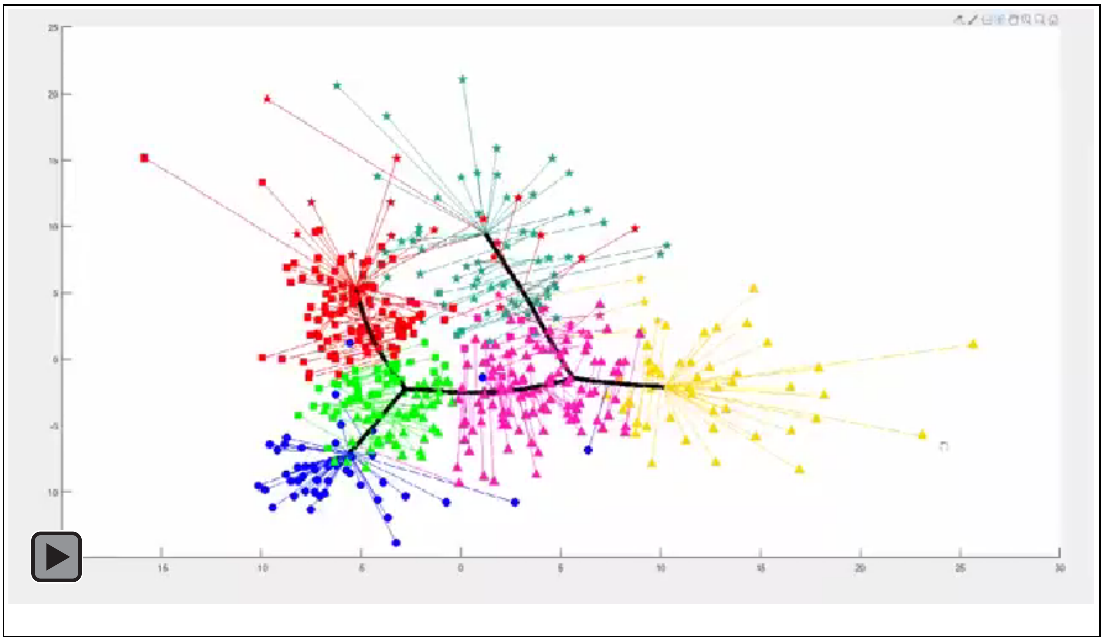

Fig B. Three-dimensional view of the model for validation cohort 1. The video is shown in the S3 Appendix.

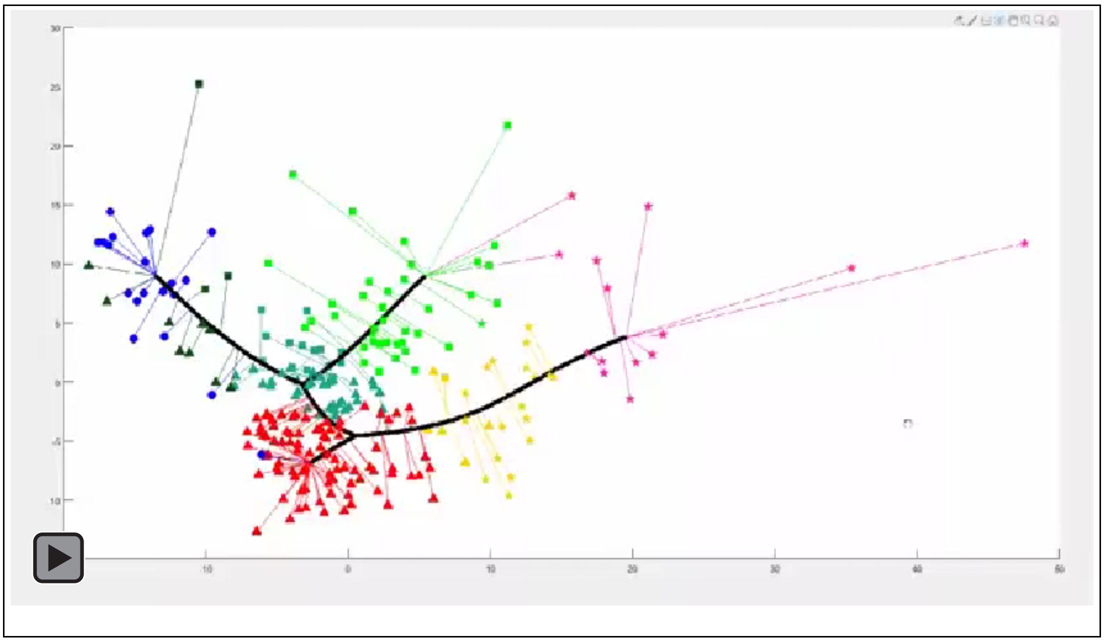

Fig C. Three-dimensional view of the model for validation cohort 2. The video is shown in the S3 Appendix.

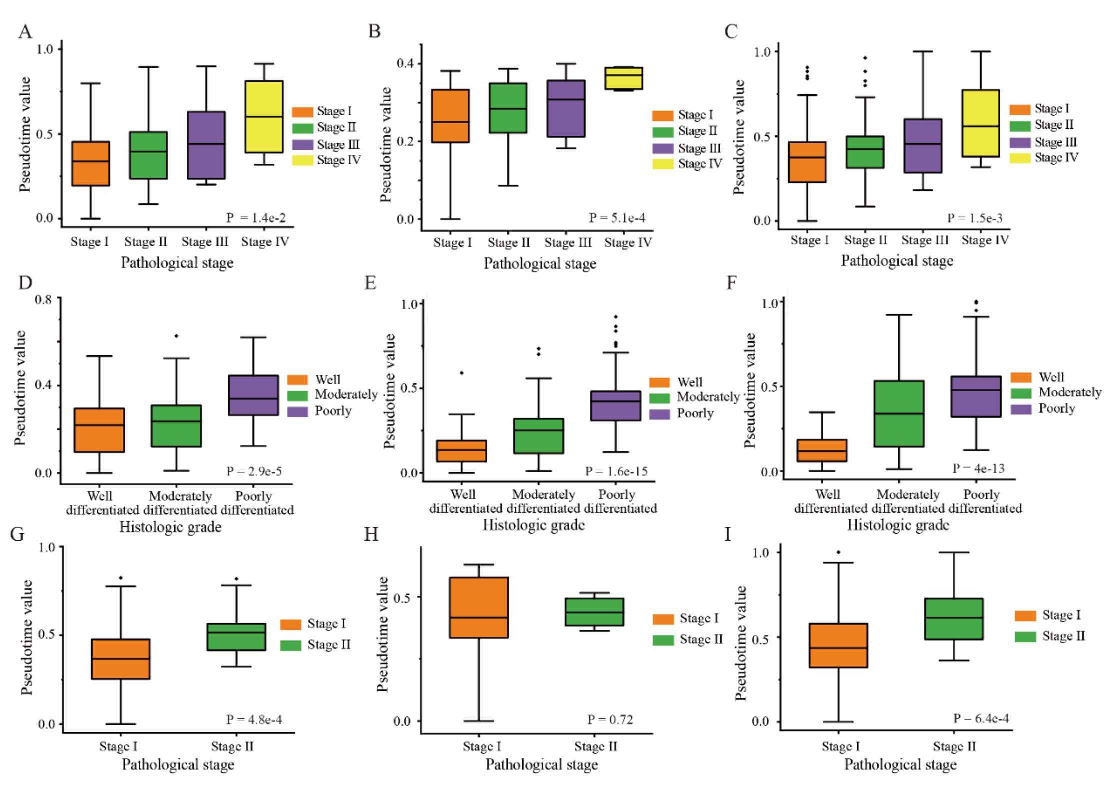

Fig D. Pseudotime value showed significant difference in distinct pathological stages for 3 different branches of three cohorts: Normal to PI (A, D, G), Normal to TRU (B, E, H), Normal to PP (C, F, I). (A-C) Three branches for TCGA-LUAD. (D-F) Three branches for validation cohort 1. (G-I) Three branches for validation cohort 2.

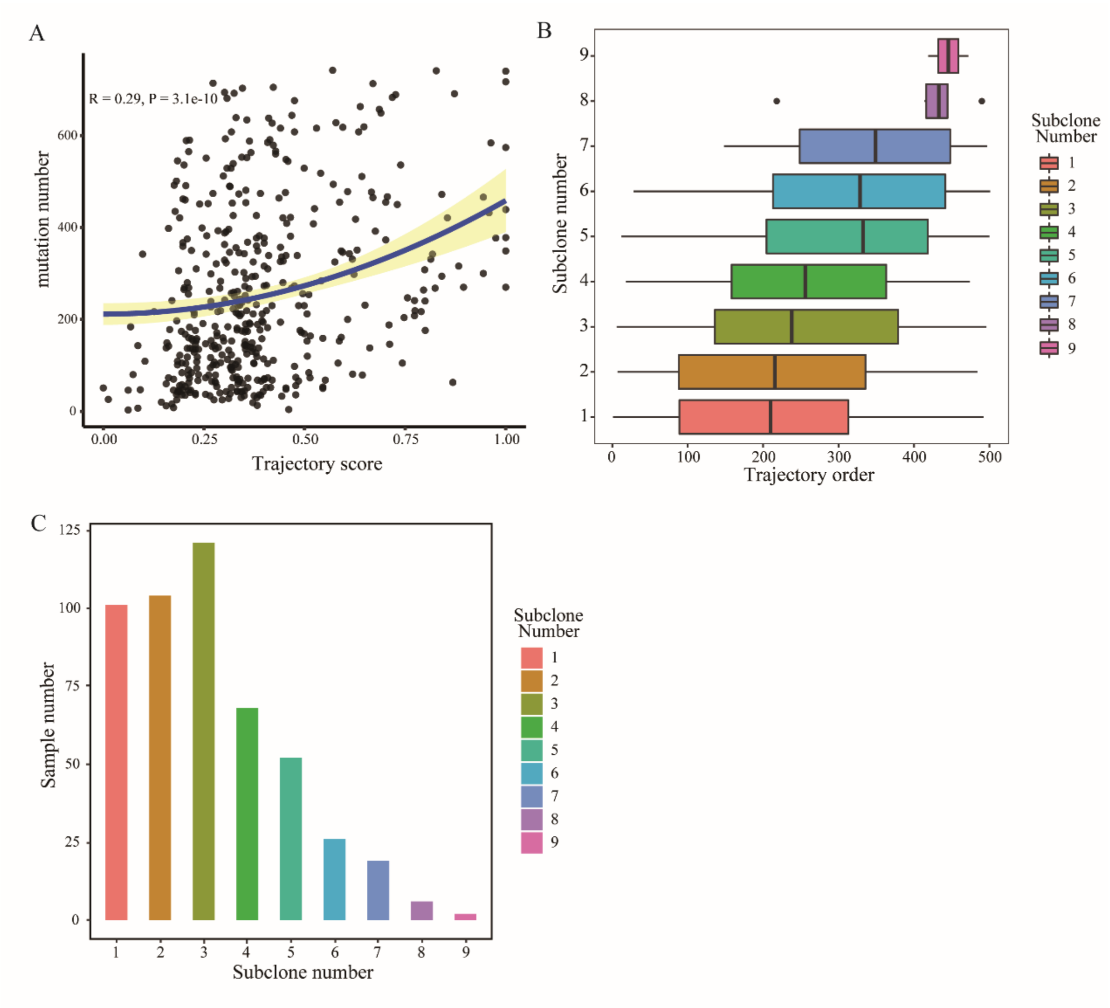

Fig E. (A) Association between trajectory score and the number of mutated genes. (B) The relationship between the number of subclones and the progression of LUAD. The number of subclones significantly increased with the progression of LUAD. (C) The sample number included in different subclone number group. This result showed that 3 subclones may more common in LUAD patients.

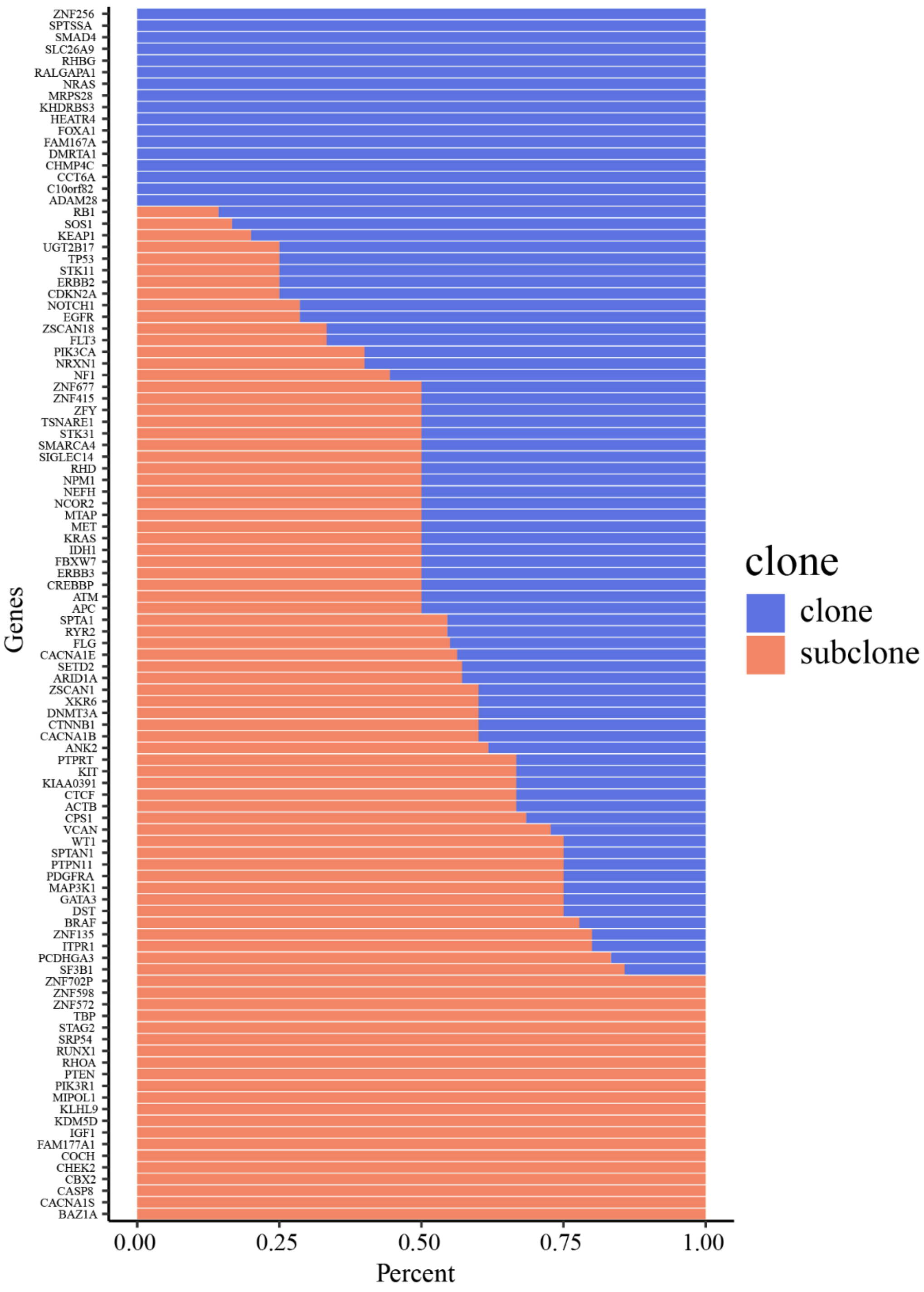

Fig F. The proportion of clone and subclone for genes in normal-PI branch.

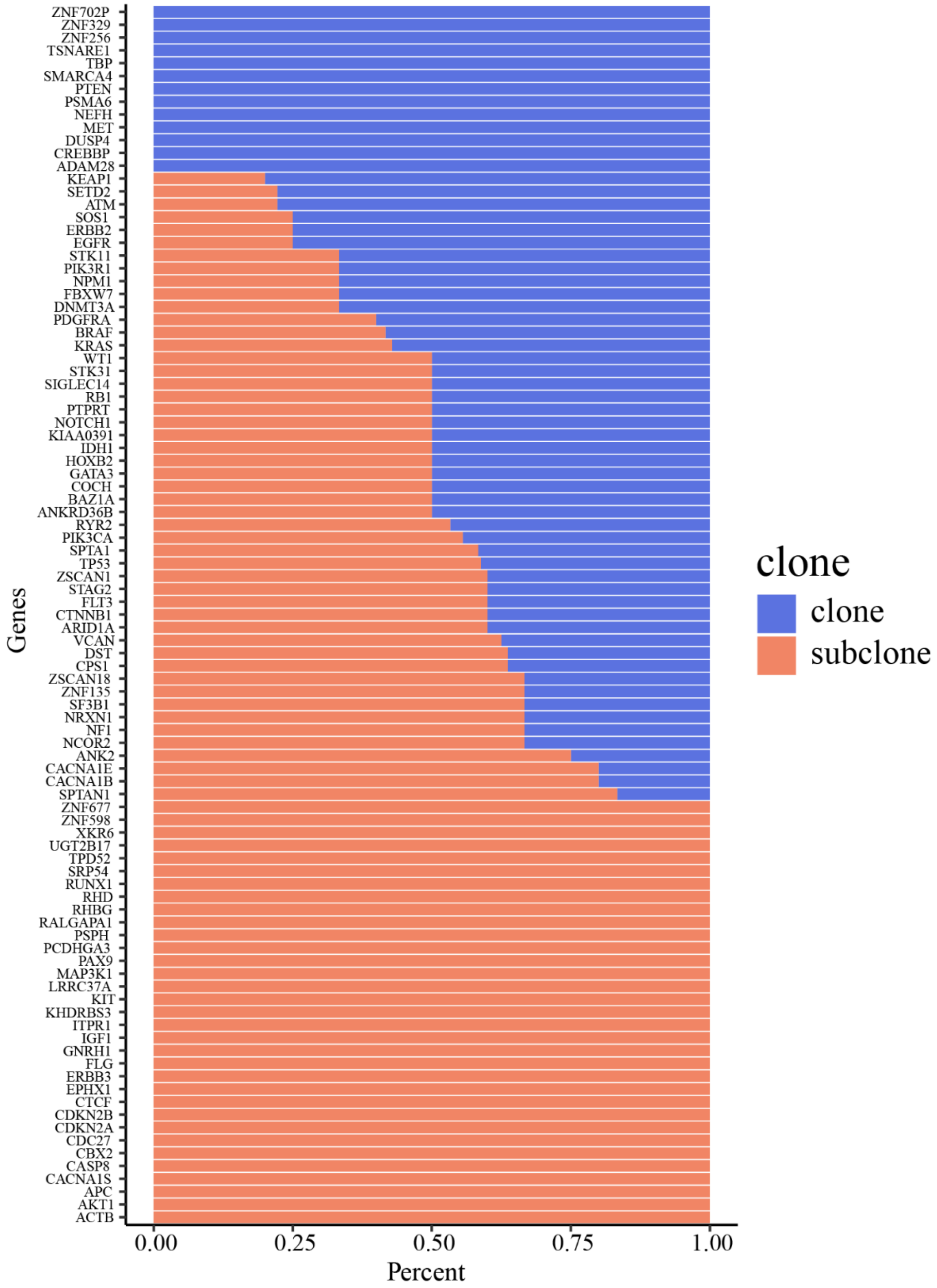

Fig G. The proportion of clone and subclone for genes in normal-TRU branch.

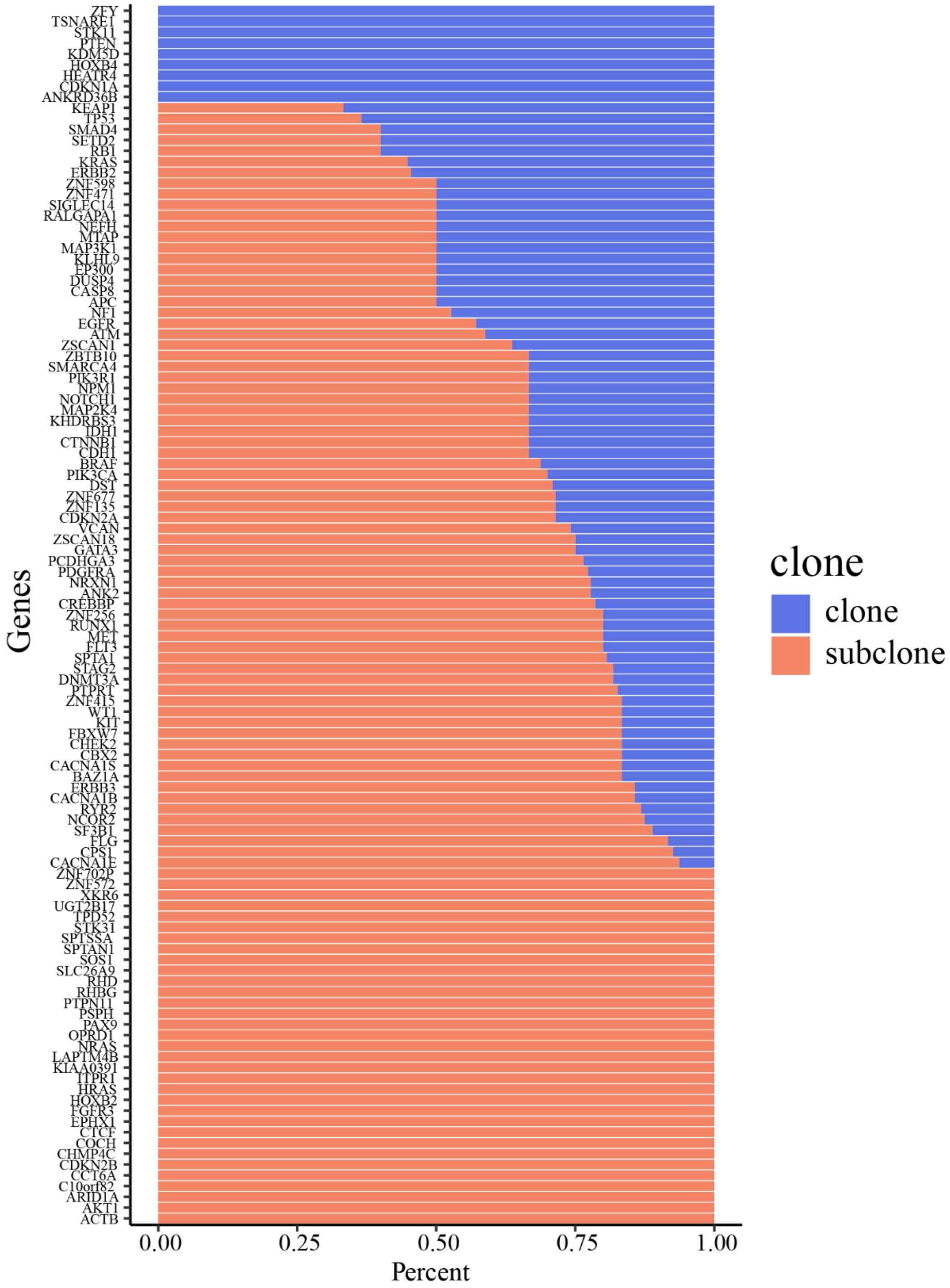

Fig H. The proportion of clone and subclone for genes in normal-PP branch.

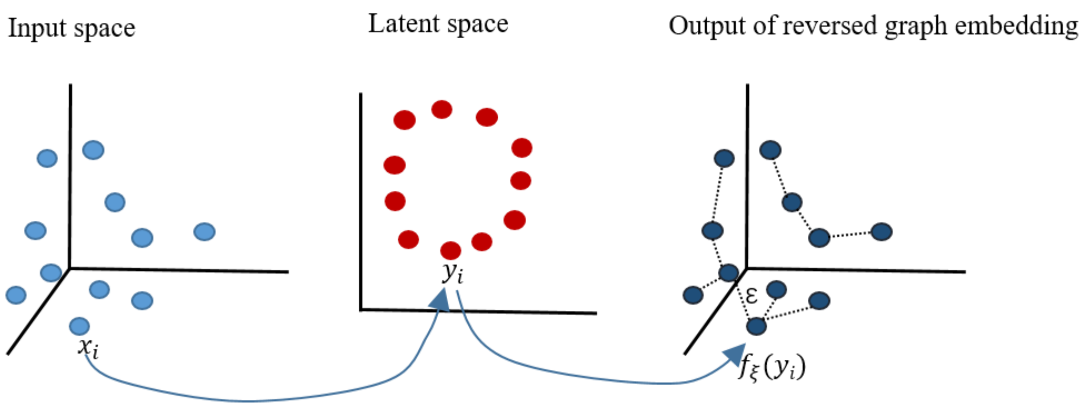

Fig I. A cartoon illustrating the reversed graph embedding method. Each point x_i in the left panel refers a sample in the input space. y_i refers this sample in the latent space. Each y_i can map to the point $f_\xi(y_i)$ by maintaining the graph structure through the reversed graph embedding. ε refers the connections between the points. By using this method, we can infer the underlying structure of high dimensional data.

Table A. Information of the four datasets used in this study.

	Dataset	Database	Platform	Sample size (LUAD + normal)
Training cohort	TCGA-LUAD	TCGA	——	533+59
Validation cohort 1	GSE68465<br>GSE10072	GEO	GPL96<br>GPL96	443+49
Validation cohort 2	GSE31210	GEO	GPL570	226+20

Table B. 314 progression-related genes selected by using MRMR and IFS method.

AC011899.9, AC109826.1, ACADL, ACRV1, ADPRH, ADRB2, AGER, AGTPBP1, ALG10, ALOX5AP, ANGPT4, ANOS1, AP1S2, AQP4, ARHGAP11A, ARHGAP18, ARHGAP31, ARHGEF26, ARPC2, ASNS, AURKA, B2M, B3GALNT1, BLM, BRI3BP, BTK, BUB1, BUB1B, BUB3, C10orf128, C10orf67, C12orf4, C14orf132, C1orf112, C1orf162, C1QA, C1QB, C1QC, CA4, CARD16, CASC5, CASP1, CASP5, CAV1, CAV2, CCNB1, CCNB2, CCR1, CCR5, CD52, CD86, CDC25A, CDK1, CENPA, CENPL, CENPU, CEP76, CFL2, CHEK1, CHEK2, CKAP2, CKAP2L, CKS1B, CLDN18, CLEC3B, CLEC7A, CLIC5, CNBP, CNTN6, CORO1C, CRYBB1, CTD-2510F5.4, CYBB, CYTH4, DAB2, DDX55, DEPDC1B, DNA2, DNAJC9, E2F7, ECSCR, ECT2, EDNRB, EMP2, EMP3, ENO1, ESCO2, EVI2B, EXO1, FABP5, FAM189A2, FAM64A, FAM72D, FAM83D, FANCI, FCER1G, FCGR1A, FCGR1B, FCGR3A, FMO2, FOXM1, FRMD3, G2E3, GAS2L3, GIMAP2, GIMAP4, GIMAP6, GIMAP7, GIMAP8, GMFG, GMNN, GNB4, GNPNAT1, GPM6A, GPN3, GPR65, GPR84, GSG2, GYPE, HAUS2, HAUS3, HAVCR2, HELLS, HJURP, HLA-E, HMGA1, HMGN4, HMMR, HPS5, HSPD1, HYLS1, IL4I1, IRAK4, ITLN2, KCTD12, KIAA0101, KIAA1524, KIF14, KIF18A, KIF23, KNSTRN, KPNA2, LACTB, LAMP3, LANCL1-AS1, LAPTM5, LAT2, LCP1, LDHA, LGI3, LINC00656, LINC00961, LINC00968, LINC01150, LMO2, LPXN, LRRC25, LY86, M6PR, MAPK6, MARCO, MARS, MCEMP1, MCM8, MGME1, MITD1, MND1, MNDA, MOB1A, MRC1, MSR1, MTHFD2, MYO7A, NAGK, NCAPG, NCF2, NCKAP1L, NCKAP5, NECAB1, NEDD1, NEIL3, NEMP1, NLN, NLRC4, NOP56, NUF2, NUSAP1, OIP5, OLR1, OTUD1, P2RX7, P2RY1, PAICS, PAK1, PARPBP, PDK1, PECAM1, PIF1, PILRA, PLEK, PLEKHO1, PLEKHO2, PPAT, PPM1M, PRC1, PRKG2, PRR11, PSAT1, PTGES3, PTPN21, PTPN7, PTPRO, PTRF, PTTG1, QKI, RAB8B, RACGAP1, RAD51, RAD51AP1, RAD54B, RASGEF1B, RCC1, RDM1, RFC4, RGCC, RHNO1, RNASE6, RP11-121A8.1, RP11-141J13.5, RP11-253E3.3, RP11-287F9.2, RP11-35J10.7, RP11-371A19.2, RP11-673E1.3, RP11-8L8.2, RP5-826L7.1, RPAP3, RPE, RS1, RTKN2, RXFP1, SAMHD1, SDCBP, SDPR, SELPLG, SENP1, SET, SFTPC, SFXN1, SGCG, SGO2,

SH3BGRL, SH3GL3, SHMT2, SIGLEC22P, SIRPB1, SKA3, SLC2A1, SLC2A5, SLC31A2, SLC39A8, SLC7A7, SLC8A1, SMC2, SNRPA1, SNX10, SPATS2, SPC24, SPDL1, SPI1, SPN, SPOCK2, SPP1, SRPK1, STAC3, STAT3, STIL, STX11, SUV39H2, TACC3, TDP1, TEK, TICRR, TIGAR, TIMELESS, TLR1, TLR7, TLR8, TM6SF1, TMEM140, TMEM177, TMEM182, TNFAIP8L2, TNFSF13B, TNNC1, TOPBP1, TPI1, TPX2, TRIM59, TRIM69, TTK, TTL, TUBA1B, UBASH3B, UBE2L6, UBE2T, UNG, VIM, VTA1, WDR75, WWC2, XPOT, ZNF695, ZWILCH

Table C. SNP loci associated with inferred trajectory based on GWAS analysis.

SNP ID	Gene	Chromosome	Position	P-value
rs10734200	PARVA	11	12531927	6.69e-11
rs9478337	SYNE1	6	152573937	9.45e-11
rs12571387	ADAM12	10	126081293	3.03e-08
rs12556027	HDX	X	84474374	4.23e-08
rs7154051	NPAS3	14	33534187	1.19e-07
rs9564321	PCDH9	13	66545709	3.20e-07
rs1569094034	DSCAM	21	40378277	5.54e-07
rs2219753	TTC39B	9	15189047	6.87e-07
rs9878766	FHIT	3	60791849	7.73e-07
rs814409	MSRA	8	10075856	8.21e-07
rs12673949	HIBADH	7	27561015	9.43e-07
rs7029746	VLDLR-AS1	9	2472254	1.15e-06
rs17107140	TNKS2	10	91840723	1.32e-06
rs17107019	FAF2P1	10	91766805	1.58e-06
rs11207010	DAB1	1	57337214	2.21e-06
rs1484057181	SPSB4	3	141056011	2.47e-06
rs41373946	GRIA1	5	153604920	2.62e-06
rs6651478	PSD3	8	19081848	2.64e-06
rs6735919	CSRNP3	2	165632556	4.15e-06
rs9866825	LMCD1-AS1	3	8209103	7.20e-06
rs1055760305	NDST4	4	114869924	7.67e-06

Table D. All available targetable genes and target drugs according to NLMT and MATCH.

Target Genes	Drugs	
	NLMT	MATCH
FGFR3	AZD4547	AZD4547
STK11	AZD2014	——
KRAS	Palbociclib	——
NF1	Selumetinib<br>Docetaxel	Trametinib
NRAS	Selumetinib<br>Docetaxel	Binimetinib
PIK3CA	AZD5363	Taselisib
AKT1	AZD5363	AZD5363
EGFR	AZD9291	Afatinib<br>AZD9291
ERBB2	——	Afatinib
MET	Crizotinib	Crizotinib
BRAF	——	Dabrafenib<br>Trametinib
PTEN	AZD5363	GSK2636771
KIT	——	Sunitinib

Table E. Soft agar colony formation in several human LUAD cell lines. The colony number of human BUB1B siRNA SMRTpool (siBUB1B) were shown as relative values normalized to controls (siNC).

Human LUAD cell lines	siNC<br>mean (SEM)	siBub1b<br>mean (SEM)	p-value
LKPH2	1.00 (0.06)	0.13 (0.01)	<0.01
LKP9	1.00 (0.07)	0.21 (0.07)	<0.05
H1437	1.00 (0.07)	0.32 (0.03)	<0.05
H1793	1.00 (0.12)	0.22 (0.07)	<0.05
H2126	1.00 (0.07)	0.33 (0.15)	<0.05
H838	1.00 (0.10)	0.27 (0.02)	<0.01

HCC827	1.00 (0.15)	0.30 (0.19)	0.1
H1299	1.00 (0.09)	0.23 (0.04)	<0.05
H358	1.00 (0.10)	0.16 (0.06)	<0.05
H1792	1.00 (0.05)	0.14 (0.03)	<0.01
H23	1.00 (0.24)	0.16 (0.09)	0.09
H2009	1.00 (0.11)	0.25 (0.05)	<0.05
A549	1.00 (0.17)	0.10 (0.04)	<0.05
H1944	1.00 (0.15)	0.12 (0.11)	<0.05
H460	1.00 (0.23)	0.14 (0.02)	0.07
